# Retraction: The approaches and methods of music psychology in the relationship between music emotion and cognition in music teaching activities

**DOI:** 10.3389/fpsyg.2024.1491672

**Published:** 2024-09-13

**Authors:** Frontiers Editorial Office

The journal retracts the October 04 2022 article cited above.

Following concerns regarding the originality of the article, an investigation was conducted in accordance with Frontiers' policies. It was found that the complaint was valid and that the article should be retracted, because of an unacceptable level of similarity with a PhD thesis published by Zhang M. (2017) in the China National Knowledge Infrastructure (CNKI).

This retraction was approved by the Chief Editor of Frontiers in Educational Psychology and the Editor-in-Chief of Frontiers in Psychology. The authors did agree to this retraction.

Frontiers would like to thank the concerned reader who contacted us regarding the published article.

